# Gut Microbial Composition and Diversity in Four Ophiuroid Species: Divergence Between Suspension Feeder and Scavenger and Their Symbiotic Microbes

**DOI:** 10.3389/fmicb.2021.645070

**Published:** 2021-03-19

**Authors:** Yue Dong, Yixuan Li, Peiqing He, Zongling Wang, Shiliang Fan, Zhixin Zhang, Xuelei Zhang, Qinzeng Xu

**Affiliations:** ^1^MNR Key Laboratory of Marine Eco-Environmental Science and Technology, First Institute of Oceanography, Ministry of Natural Resources, Qingdao, China; ^2^Laboratory for Marine Ecology and Environmental Science, Pilot National Laboratory for Marine Science and Technology, Qingdao, China; ^3^Department of Biology, Hong Kong Baptist University, Hong Kong, China; ^4^Weihai Aquatic School, Weihai, China

**Keywords:** gut microbiota, ophiuroids, feeding type, symbiotic bacteria, functional microbiomes, Yellow Sea

## Abstract

Gut microbiota have important roles in the survival and adaptation of the host. Ophiuroids, as the worldwide dominant benthos, have ecological roles in benthic–pelagic coupling in the sea floor. However, little is known about the composition and diversity of their gut microbiota and its potential functions in benthic ecosystems. In present study, we preformed 16S rRNA sequencing and function analysis in four dominant species (*Stegophiura sladeni*, *Ophiopholis mirabilis*, *Ophiura sarsii vadicola*, and *Ophiura kinbergi*) with two feeding types (suspension feeding/herbivores and scavenger/carnivores) from the Yellow Sea, China. Results showed that 56 phyla and 569 genera of microbiota were identified among ophiuroid guts. Multivariate and diversity analyses showed that the ophiuroid gut microbiota were independent and have higher biodiversity to the sediment microbial in the Yellow Sea. Phyla Proteobacteria, Firmicutes, Tenericutes, and Bacteroidetes were the dominant bacteria, with more than 80% abundance among the four ophiuroid species. A comparison among the gut microbial compositions among four ophiuroids showed the similarity of two offshore carnivore ophiuroids (*S. sladeni* and *O. sarsii vadicola*) and variation in the dominant microbiota types of three nearshore ophiuroids (*S. sladeni*, *O. mirabilis*, and *O. kinbergi*). The functional analysis revealed the significant differences of the environment-related expression in *S. sladeni* gut microbiota between nearshore and offshore environments. The Phylogenetic Investigation of Communities by Reconstruction of Unobserved States (PICRUSt) functional annotation showed the significant divergence of metabolism pathways between two nearshore species, the herbivores *O. mirabilis* and carnivores *S. sladeni*, such as the Lipid metabolism, Carbohydrate metabolism, and Metabolism of cofactors and vitamins. The homolog search and phylogenetic analysis identified the first gut symbiotic *Candidatus* Hepatoplasma in *S. sladeni* with important roles for the nutrient metabolisms. Overall, our study reported the comprehensive data of ophiuroid gut microbiota, while the functional microbiome provides insight into the physiology and environmental adaptation in ophiuroids.

## Introduction

Ophiuroidea (brittle stars), with 2,064 known species from 16 families, are the largest class of Echinodermata (Geraldi et al., 2017). They play an important ecological role in food webs (Wu and Shin, 1997; Harris et al., 2009; Enoksen and Reiss, 2017) and nutrient recycling in the marine benthic ecosystems (Lebrato et al., 2010; Ravelo et al., 2015). The Ophiuroids possess two feeding types: suspension feeding and scavenger. Brittle stars from families Gorgonocephalidae and Amphiuridae are primarily suspension feeding on organic detritus, plankton, and bacteria (Iken et al., 2001). Scavenger ophiuroids, including Ophiopyrgidae and Ophiacanthidae, feed on dead organisms and small animals (usually dead), including crustaceans, mollusks, and worms (Adarsha et al., 2018). In addition, the assemblage of ophiuroid populations is a common phenomenon in different habitats (Murat et al., 2016), given that they could enhance the process of benthic–pelagic coupling in the sea floor (Geraldi et al., 2017).

The gut microbiota plays key roles in nutrient absorption, environmental adaptation, and anti-pathogens of the host animals (Sugita et al., 1991; Cho and Blaser, 2012; Zhang et al., 2016). The habitat environment (Trabal et al., 2012; Pierce et al., 2016; Nguyen et al., 2020), host feeding habits, and life stages (Tanaka et al., 2004; Li et al., 2019) have effects on the gut microbial community. Studies on the microbiome of echinoderms revealed that gut microbiota in sea cucumber (Zhang et al., 2012, 2013), starfish (Lee et al., 2018), and sea urchin (Hakim et al., 2016) can enhance the digestion and provide missing nutrients from diets to the host. However, the role of gut microbiota still remains unclear, which hinders the understanding of physiologies, ecologies, and life histories of ophiuroids. In addition, the composition of gut microbiota may have divergences among species with different feeding types (Guo et al., 2020). Some echinoids have evolved gut symbiotic bacteria for acquisition of essential nutrients (Temara et al., 1993; Becker et al., 2009). In ophiuroids, symbiotic bacteria have identified on larvae and adult subcuticle to facilitate the uptake of free amino acid from the ambient environment (Walker and Lesser, 1989; Morrow et al., 2018), while the gut symbiont has not been reported yet.

In this study, we focus on the gut microbiota communities of ophiuroids that lived in the Yellow Sea, a marginal sea between China and Korean Peninsula with hydrological phenomena and high biodiversity (Li, 2011). Four dominant microbenthic ophiuroids, including *Stegophiura sladeni*, *Ophiopholis mirabilis*, *Ophiura sarsii vadicola*, and *Ophiura kinbergi* (Peng et al., 2017), were selected for 16S rRNA sequencing of their gut microbial communities, which covered two feeding types, scavengers (*O. sarsii vadicola*, *O. kinbergi*, and *S. sladeni*; Harris et al., 2009) and suspension feeder (*O. mirabilis*; Yu et al., 2019). The alpha diversity and multivariate analysis revealed the spatial variation among nearshore and offshore ophiuroids, and their surrounding sediments. The diversity and functional analyses of microbiome indicated significant variation between scavenging and suspension-feeding ophiuroids. Integration of metagenomic sequencing and functional-inference-based approaches provided insight into the metabolic and environmental adaptation from gut communities, which facilitate their survival and organic matters recycling in benthic ecosystems.

## Materials and Methods

### Sample Collection

**FIGURE 1:**
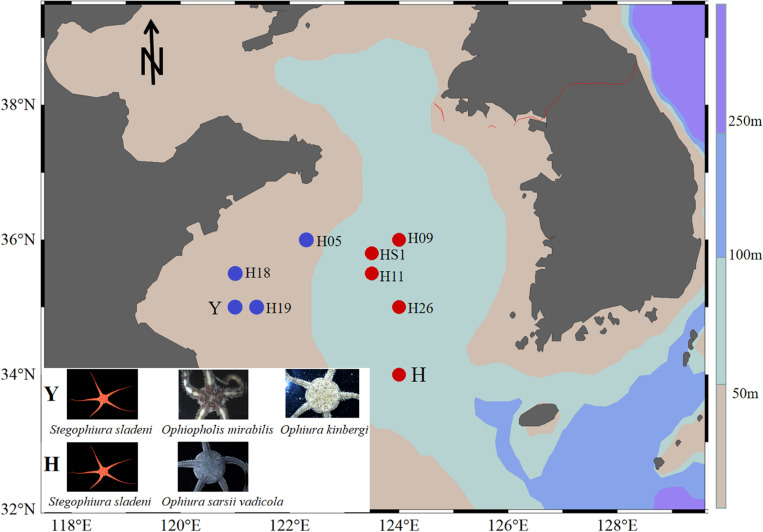
Location of sampling sites in the southern Yellow Sea. Ophiuroids were collected from nearshore (station Y) with blue color and offshore (station H) with red color. Sediment stations were H05, H18, H19, H09, HS1, H11, and H26 (Zhang et al., 2019).

Ophiuroid specimens were collected by bottom trawling from the Yellow Sea: offshore (water depth > 50 m) station H (34°00′N, 124°00′E, 80-m depth) for *S. sladeni* and *O. sarsii vadicola* in March 2018 and nearshore station Y (35°00′ N, 121°00′ E, 16-m depth) for *S. sladeni*, *O. mirabilis*, and *O. kinbergi* in October 2018 (Figure 1). All specimens were immediately fixed in 95% ethanol and stored at −20°C. After cruises, specimens were transferred to the First Institute of Oceanography, Ministry of Natural Resources, for further analysis. To reduce the contamination by environmental bacteria, the ophiuroids were rinsed with Milli-Q water before dissection. The oral shield was removed, and the gastric contents were sampled under a stereomicroscope. All gut contents were stored in a freezer at −20°C until use for DNA extraction.

### Total DNA Extraction and 16S rRNA Sequencing

To understand the gut microbiota, genomic DNA was extracted from the gut contents of 23 ophiuroid specimens (nearshore: four *S. sladeni*, five *O. mirabilis*, and five *O. kinbergi*; offshore: five *S. sladeni* and four *O. sarsii vadicola*) and used for sequencing on the V3–V4 region of the microbial 16S rRNA gene. In brief, total microbial community DNA was isolated using the DNeasy Blood & Tissue Kit (Qiagen, Germany) following the manufacturer’s protocol. DNA quality was assessed using agarose gel electrophoresis and a Qubit fluorometer (Thermo Fisher Scientific, Waltham, MA, United States). The V3–V4 hypervariable region of 16S rRNA gene were amplified using the forward primer 338 F (5′-GTACTCCTACGGGAGGCAGCA-3′) and the reverse primer 806 R (5′-GTGGACTACHVGGGTWTCTAAT-3′). The PCR contained 30 ng of DNA template (30 ng), 1 μl each of 5 mol/L of primers, 3 μl of bovine serum albumin (BSA) (2 ng/μl), 12.5 μl of 2 × Taq Plus Master Mix, and 7.5 μl of ddH_2_O in a volume of 25 μl. PCR amplification was performed with the following program: pre-denaturation at 94°C for 5 min; 30 cycles of denaturation at 94°C for 30 s, annealing at 50°C for 30 s, and an extension at 72°C for 60 s; and a final extension at 72°C for 7 min. The PCR products were sequenced using an Illumina MiSeq sequencer to produce 300-bp paired-end reads by Allwegene Technology (Beijing, China).

### Data Filtration and Amplicon Sequence Variant Clustering

The raw sequencing reads were filtered by removing adapters using Cutadapt software (Marcel, 2011; Callahan et al., 2016). The following taxonomic analyses were performed using the R script implemented in dada2 v1.16.0 packages (Callahan et al., 2016) with the default parameters. The clean reads were further quality filtered, dereplicated, and merged using the R script filterAndTrim, derepFastq, and mergePairs, respectively. The makeSequenceTable command was used to construct an amplicon sequence variant (ASV) table. Potential chimeras were removed using the removeBimeraDenovo command. ASVs were annotated compared with the Silva SSU rRNA database (version 138) for taxonomic classification (Benjamin, 2017) using assignTaxonomy command.

To compare the microbial community in ophiuroid gut and in the sediment, 16S rRNA sequences of the Yellow Sea sediments (three stations near the Y site, four stations near the H site) were downloaded from Zhang et al. (2019; Figure 1). The sequence filtering, clustering, and annotation were performed the same as above. The gut microbiota of ophiuroids and sediment had different ASV numbers and compositions. The results of species-level annotation showed that the proportion of unknown species was 97.5%, so we performed downstream analysis based on the genus and above level. The functional profiles of the gut microbial communities based on 16S rRNA sequences were annotated using Tax4Fun (Aßhauer et al., 2015) and Phylogenetic Investigation of Communities by Reconstruction of Unobserved States (PICRUSt) (Langille et al., 2013) with Kyoto Encyclopedia of Genes and Genomes (KEGG) Orthology (KO) (Minoru et al., 2015). To compare functional differences among samples, abundances of predicted functional pathways were normalized sequencing depth as percentages of the total number of predicted functions from the KO database. Three sets of pairwise comparison analysis were performed for the bacterial function pathways of ophiuroid gut microbial communities, including *S. sladeni* and *O. mirabilis* from the nearshore environment, *S. sladeni* and *O. sarsii vadicola* from the offshore environment, and *S. sladeni* from both two environments. Statistical Analysis of Metagenomic Profiles (STAMP) analysis indicated the significant variations in KOs with expression levels above 1% contribution.

### Phylogenic Analysis

To investigate any possible gut symbionts, the ASVs of gut microbiota were BLASTn search against GenBank database with threshold of identity >80% and E-value of 1e−20. Among ASVs, only genus *Candidatus* Hepatoplasma showed homology to known gut symbionts. To further elucidate the microbial candidates, eight ASVs of *Ca*. Hepatoplasma, their top BLAST hit sequences, and nine 16S rRNA symbiont sequences [from Cheng et al. (2019) and National Center for Biotechnology Information (NCBI)] were aligned by ClustalW and manually trimmed. Maximum likelihood (ML) and neighbor joining (NJ) were selected for phylogenetic analysis with MEGA v7 (Kumar et al., 2016). The best model of Kimura two-parameter selected by model test in MEGA and 1,000 bootstrap replications were used.

### Statistical Analysis

Alpha diversity statistics were calculated for diversity metrics based on Simpson index and Shannon index. The Mann–Whitney *U* tests were used to evaluate the differences among categories in R. Principal component analysis (PCA) reflects the difference and distance between samples by analyzing ASVs with Origin 2018 software at the genus level (Wang et al., 2012). Analysis of similarity (ANOSIM) was used to evaluate the differences in the microbiota communities among the gut of ophiuroids, and between ophiuroids’ gut and benthic sediment (Clarke, 1993). Similarity percentage analyses (SIMPERs) were used to identify the contribution of each taxon to the community dissimilarity (Clarke, 1993). Permutational multivariate analysis of variance (PERMANOVA) revealed the amount of variation with respect to both the microbiota in the gut of ophiuroids and sediment (Anderson, 2014). Those three analyses were performed using PRIMER version 6 and PERMANOVA + software. The linear discriminant analysis (LDA) effect size (LEfSe) analysis was conducted by the Galaxy website under default parameters^1^ to evaluate the significance of differences (i.e., biomarkers) at the phylum, class, order, family, and genus levels in five ophiuroid groups. In addition, the abundance of KEGG pathways over 1% was selected to calculate the ANOVA between two selected ophiuroids by using the STAMP software (Parks and Beiko, 2010).

## Results

### Amplicon Sequence Variant Analysis

**FIGURE 2:**
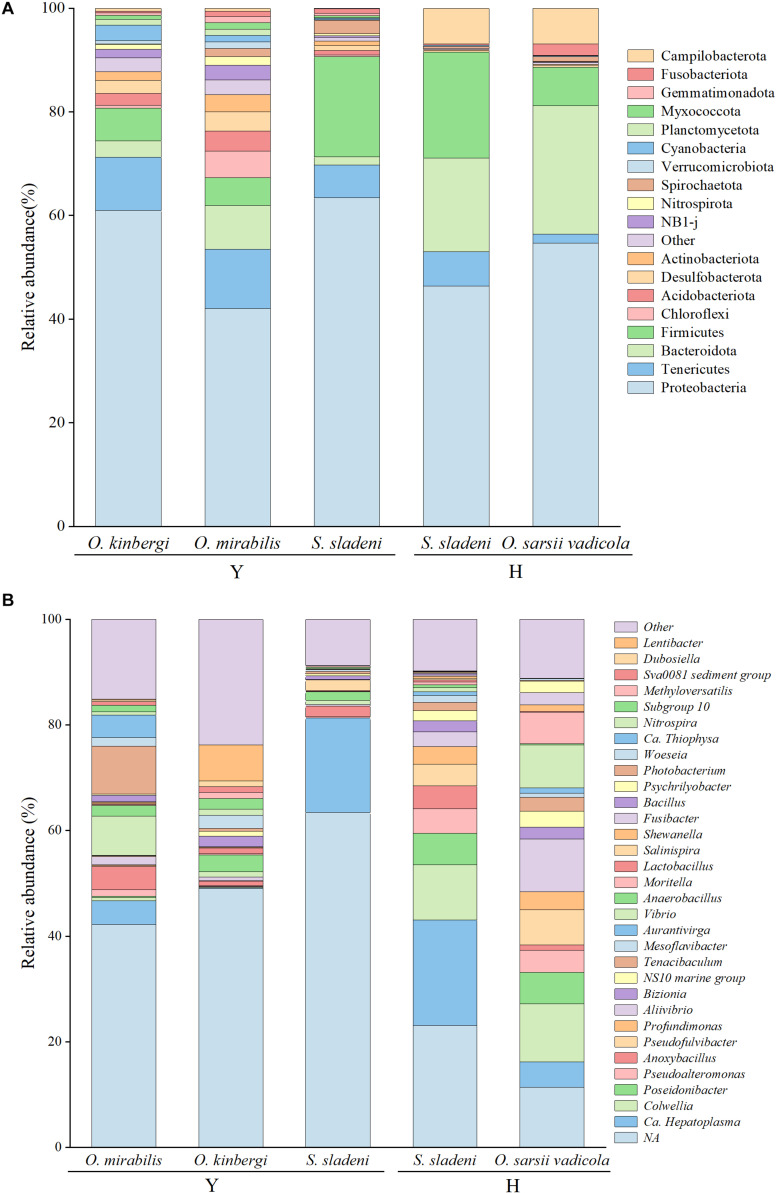
Taxonomic abundance of gut microbiota at the phylum level **(A)** and genera level **(B)** in ophiuroid species from the Yellow Sea, China. Other, the taxonomic groups with abundances less than 1%; Y, the nearshore environment; H, the offshore environment.

**FIGURE 3:**
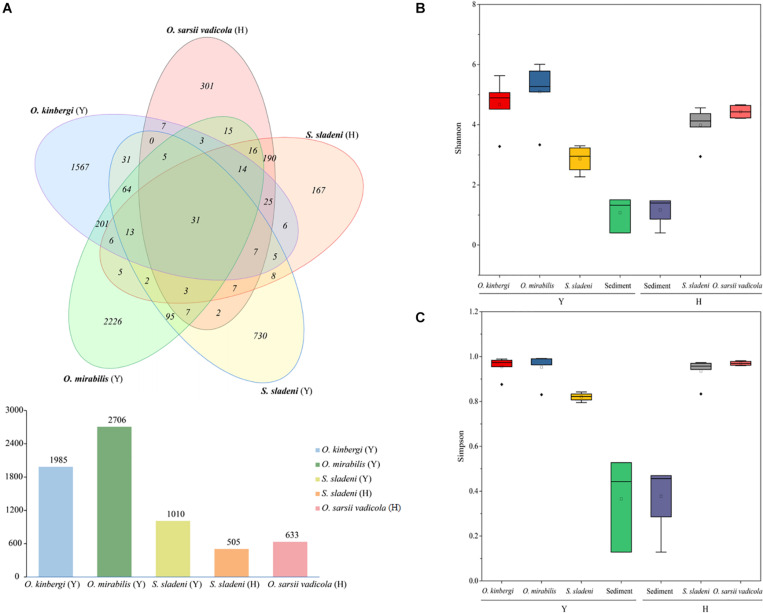
Amplicon sequence variant (ASV) Venn diagram of gut microbiota of ophiuroids and alpha diversity analyses of the ophiuroid gut and sediment microbial communities. **(A)** ASVs of ophiuroids’ gut microbiota are depicted in a Venn diagram. Alpha diversity was calculated using **(B)** the Shannon index and **(C)** the Simpson index. Statistical analysis was conducted on alpha diversity using Mann–Whitney *U* tests. ns, not significant; *p* > 0.05. Y, the nearshore environment; H, the offshore environment.

After quality filtering, 586,254 clean reads (9,101–36,825 reads per sample) were clustered in 5,759 ASVs (505–2,706 per sample) from 23 nearshore and offshore ophiuroids (Supplementary Appendix A1). The ASVs were well annotated from the phylum and genus levels (Figures 2A,B). Based on the composition analysis of the gut microbiota at the ASV level (Figure 3A), 31 ASVs were common to ophiuroids’ gut microbiota in different areas. The number of unique ASVs in each community was counted, with 730 unique ASVs in the *S. sladeni*, 1,567 in the *O. kinbergi*, and 2,226 in the *O. mirabilis* from the nearshore environment, and 167 in the *S. sladeni* and 301 in the *O. sarsii vadicola* from the offshore environment.

### Gut Microbiota Composition

#### The Gut Microbiota of Ophiuroids From Nearshore Environment

The identified gut microbiota of three ophiuroid species *S. sladeni*, *O. mirabilis*, and *O. kinbergi*, from nearshore included 56 phyla and 540 genera. In the phylum level, Proteobacteria was the highest abundance microbiota among the *S. sladeni*, *O. kinbergi*, and *O. mirabilis* in 63.4%, 60.9%, and 42.0%, respectively (Figure 2A). The phyla Firmicutes, Tenericutes, and Bacteroidetes were also the dominant microbiota in the three species, with various abundance among species. There were some species-specific abundance phyla in three ophiuroids, such as Spirochaetota (2.6%) in *S. sladeni*, Cyanobacteria (3.0%) in *O. kinbergi*, and Chloroflexi (5.1%) in *O. mirabilis*.

**FIGURE 4:**
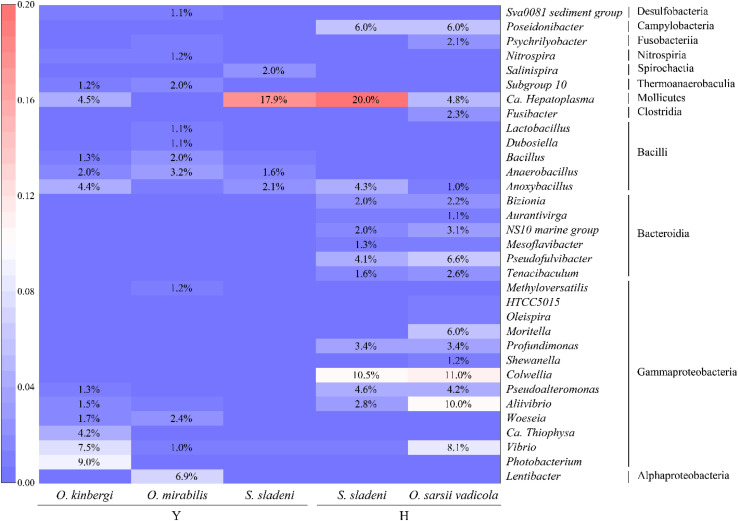
The heatmap of relative abundance of gut microbiota at the genus level (>1%) in ophiuroid species from the Yellow Sea. Y, the nearshore environment; H, the offshore environment.

At the genus level, more detailed divergences were found in the three species (Figures 2B, 4). The genus *Candidatus* Hepatoplasma was commonly found in all three ophiuroids with different abundance: 17.9% in *S. sladeni*, 0.2% in *O. mirabilis*, and 4.5% in *O. kinbergi*. *Lentibacter* was the most dominant group in *O. mirabilis* (6.9%), while only few (<0.01%) were detected in another two ophiuroids. In the gut of *O. kinbergi*, *Photobacterium* (9.1%) and *Vibrio* (7.5%) were much higher than those in *S. sladeni* (0.5 and 0.8%) and *O. mirabilis* (0.6 and 1.0%).

#### The Gut Microbiota of Two Ophiuroids From Offshore Environment

The gut microbiota of *S. sladeni* and *O. sarsii vadicola* from offshore were identified, including 31 phyla and 156 genera. Consistent with the gut microbial composition of ophiuroids from the nearshore area, Proteobacteria was the dominant phylum in *S. sladeni* and *O. sarsii vadicola*, accounting for 46.4 and 54.6%, respectively (Figure 2A). Firmicutes (20.5%) was the second highest group in the gut of *S. sladeni*, followed by Bacteroidota (18.1%), Campilobacterota (6.9%), and Tenericutes (6.7%). For *O. sarsii vadicola*, the gut microbiota were also dominated with Bacteroidota (24.8%) and Firmicutes (7.4%), Campilobacterota (6.9%), Fusobacteriota (2.1%), and Tenericutes (1.7%).

At the genus level, the high similarity occurred in the gut microbiota of two species (Figures 2B, 4). The genera had the similar abundance in *Colwellia* (10.5% of *S. sladeni* and 11.0% of *O. sarsii vadicola*), *Poseidonibacter* (6.0 and 6.0%), and *Pseudofulvibacter* (4.1 and 6.6%). It was worth noting that *Ca*. Hepatoplasma (20.0%) was the top dominant genus in *S. sladeni*, while its abundance was lower in *O. sarsii vadicola* (4.8%, Figure 4).

#### Alpha Diversity Analysis

The above ASV results showed that the gut microbiota in *O. mirabilis* and *O. kinbergi* were more specific than those of other species. In terms of alpha diversity (Figures 3B,C), the Simpson index and Shannon index (a measure of richness and evenness) were the highest in *O. mirabilis* (*p* > 0.05). In contrast, the Simpson index and Shannon index of nearshore *S. sladeni* were the lowest among ophiuroids (*p* > 0.05). And these indexes of sediment communities (nearshore and offshore) were lower than those of ophiuroids (*p* > 0.05). The alpha diversity metrics of the gut communities were significantly more diverse (Mann–Whitney *U* tests, *p* > 0.05) than the sediment microbiome.

#### Multivariate Statistical Analysis Among Ophiuroids’ Gut Microbiota and Sediment Microbiome

**FIGURE 5:**
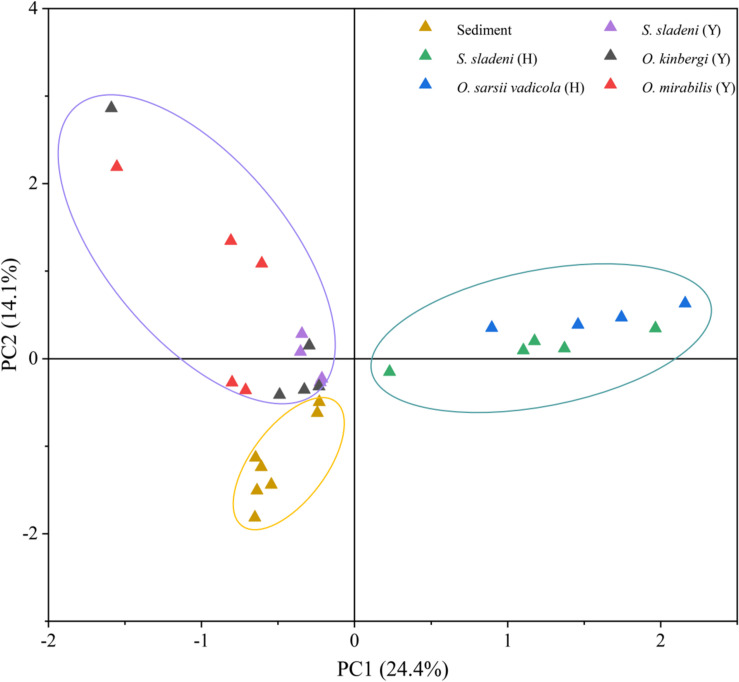
Principal component analysis (PCA) of gut microbiota in ophiuroids and sediment microbiome under genus level. Y, the nearshore environment; H, the offshore environment.

Principal component analysis revealed the strong clustering of nearshore ophiuroids, offshore ophiuroids, and sediment microbiomes (Figure 5). The first two axes of PCA accounted for 38.5% of variation with and the gut microbiota of ophiuroids separated from the sediment communities. The difference between offshore ophiuroids and sediment was also statically significant based on the results of ANOSIM and PERMANOVA (Supplementary Appendix A2, *p* < 0.05). Overall, the results of alpha diversity and multivariate analysis demonstrated the independence of microbial communities between benthic ophiuroids and sediment in the Yellow Sea.

**FIGURE 6:**
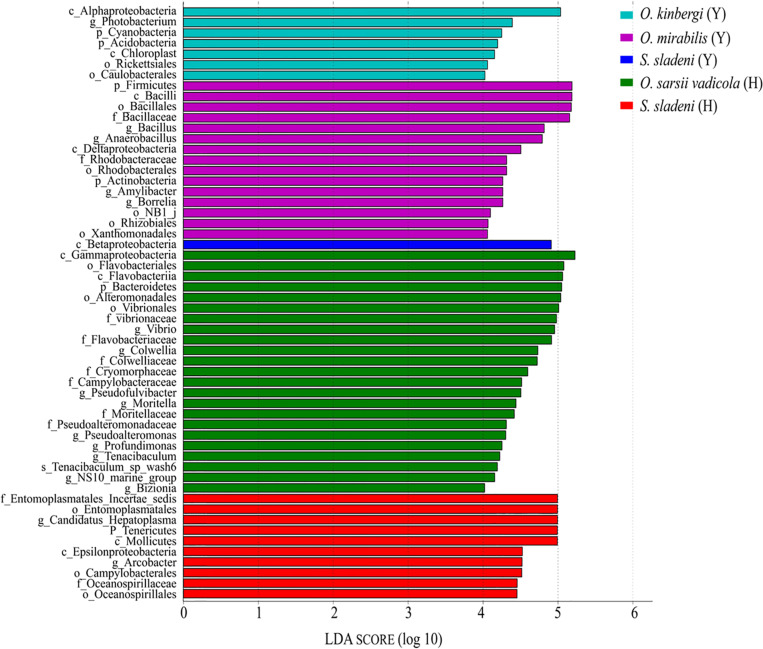
Characterization of gut microbiota in ophiuroids by LEfSe analysis and histogram of the linear discriminant analysis (LDA) scores (log 10). Y, the nearshore environment; H, the offshore environment; g, the genus; f, the family; o, the order; c, the class; p, the phylum.

The result of LEfSe analysis showed that the biomarkers in the gut microbial communities were different among five ophiuroid groups (Figure 6; Supplementary Appendix A4). In the nearshore environment, the LDA scores indicated that the class Betaproteobacteria was the largest contributor to intergroup differences in *S. sladeni*; the phylum Firmicutes and class Bacilli have significant contributions to *O. mirabilis*; the class Alphaproteobacteria was a prominent contributor to the intergroup differences in *O. kinbergi*. In the offshore environment, the class Gammaproteobacteria was the main contributor to the difference in *O. sarsii vadicola*. For *S. Sladeni*, the genus *Ca. Hepatoplasma* and higher taxonomy level of this genus were the most prominent difference contributors.

### Predictive Function of the Microbiomes

In level 2 KOs, a total of 38 and 47 pathways were annotated by Tax4Fun and PICRUSt analysis, respectively (Supplementary Appendix A5, A6). Five metabolism pathways were commonly detected in all four ophiuroids, including metabolism of cofactors and vitamins, amino acid metabolism, carbohydrate metabolism, metabolism of terpenoids, and metabolism of other amino acids.

**FIGURE 7:**
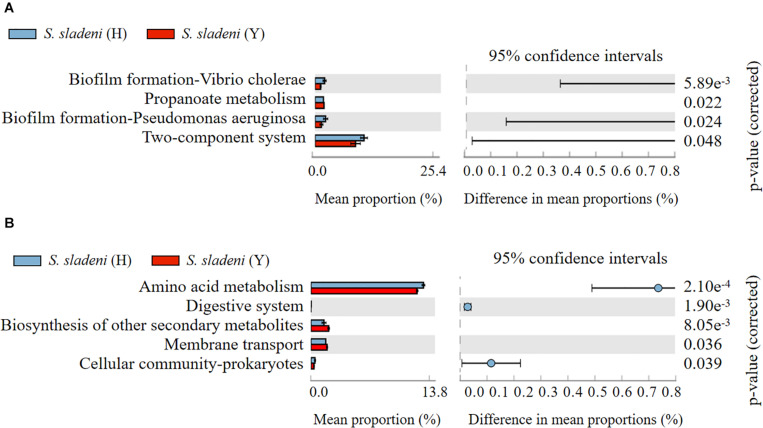
The significant expression pathways based on **(A)** Tax4Fun and **(B)** PICRUSt analysis of gut microbial community between the nearshore and offshore *Stegophiura sladeni* at Kyoto Encyclopedia of Genes and Genomes level 2.

The results of Tax4Fun showed that there was no significant difference within the groups of the nearshore and offshore ophiuroids (Supplementary Appendix A7, A8). The significance of differences in certain expression pathways was found between *S. sladeni* in the nearshore and offshore environments (*p* < 0.05, Figure 7A), while the four enriched KOs were Biofilm formation-*Vibrio cholerae*, Propanoate metabolism, Biofilm formation-*Pseudomonas aeruginosa*, and Two-component system, which belonged to three metabolic pathways Environmental Information Processing, Environmental Information Processing, and Cellular Processes, suggesting the possible interaction between the gut microbial communities and environment microbiota in *S. sladeni*. The PICRUSt analysis also showed the enriched KOs that were consistent with those results of Tax4Fun, where the five significantly different KOs were related to environmental signaling and metabolism pathways (*p* < 0.05, Figure 7B). Using an integrated Tax4Fun and PICRUSt methods, our results indicated the remarkable enrichment of functional pathways under Environmental Information Processing in *S. sladeni* gut microbiomes.

**FIGURE 8:**
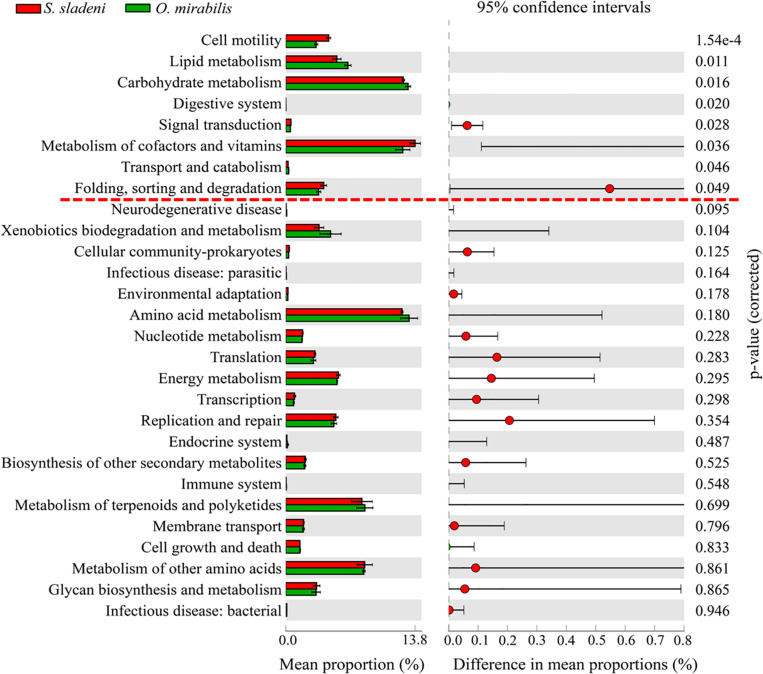
Variation analysis of gut microbial community between nearshore environment ophiuroid species *Stegophiura sladeni* and *Ophiopholis mirabilis* at Kyoto Encyclopedia of Genes and Genomes level 2 by PICRUSt. Dotted line: division of *p* < 0.05.

**FIGURE 9:**
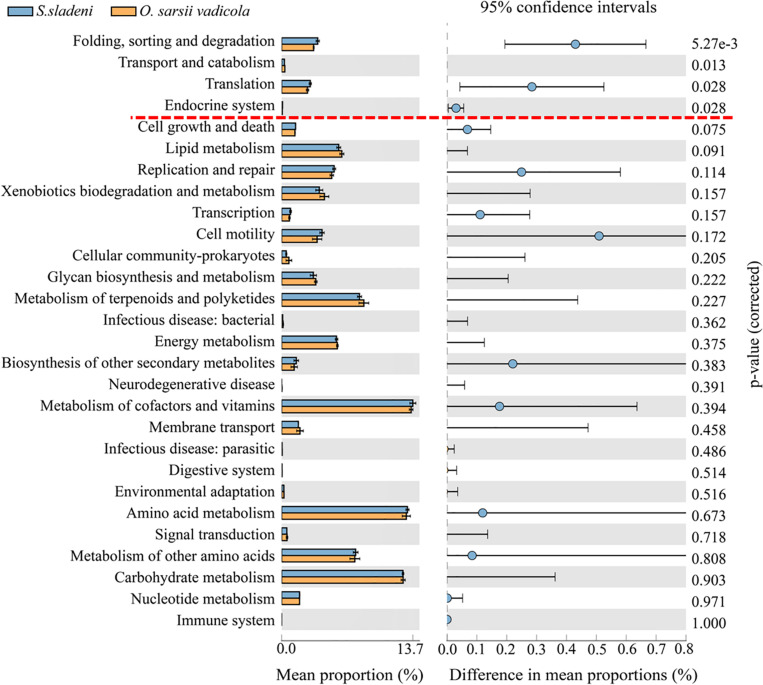
Variation analysis of gut microbial community between offshore environment ophiuroid species *Stegophiura sladeni* and *Ophiura sarsii vadicola* at Kyoto Encyclopedia of Genes and Genomes level 2 by PICRUSt. Dotted line: division of *p* < 0.05.

In the nearshore environment, the results of PICRUSt analysis showed that eight KOs were significantly divergent (*p* < 0.05) between the two species *S. sladeni* and *O. mirabilis*, mainly in Metabolism pathways (Figure 8). In the offshore environment, gut microbiota of *S. sladeni* was statistically enriched in four KOs (*p* < 0.05) including Folding, sorting, and degradation; Transport and catabolism; and Translation and Endocrine system (Figure 9).

### The Phylogenetics of Candidatus Hepatoplasma, the Mainly Symbiotic Bacteria in *Stegophiura sladeni*

**FIGURE 10:**
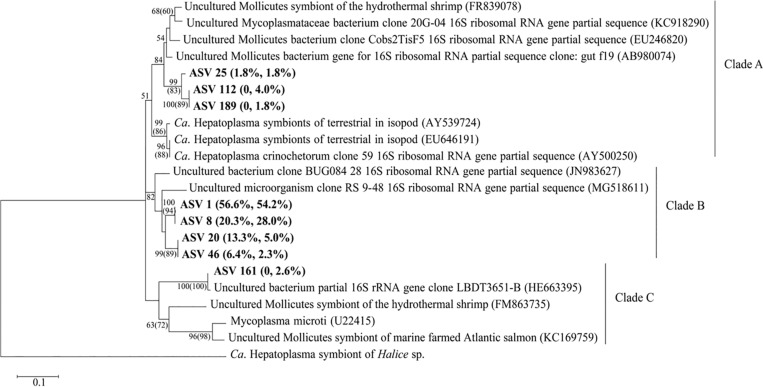
Phylogenetic analysis of *Candidatus* Hepatoplasma. The percentage represented the relative abundance in *Stegophiura sladeni* at nearshore and offshore. The bootstrap values (>50%) of relevant nodes are shown based on 1,000 replicates. The two numbers on the branch were the result of the maximum likelihood (ML), with neighbor-joining (NJ) algorithm in brackets. Sequences from this study are shown in bold.

A BLASTn search indicated that eight ASVs of genus *Ca*. Hepatoplasma from *S. sladeni* were close to bacterial symbionts of *Ca.* Hepatoplasma with identity of >80% (Supplementary Appendix A9, A10). Phylogenetic analysis of *Ca*. Hepatoplasma and their 16S rRNA homologs revealed the potential symbiotic relationship of all eight ASVs and further clustered them into three groups (Figure 10). Groups A and C, however, were specific to four *Ca*. Hepatoplasma ASVs from offshore *S. sladeni*. Given their higher abundance than nearshore *S. sladeni* (10.2 and 1.8%) and PICRUSt annotation at Amino acid metabolism and Biosynthesis of other secondary metabolites pathways, Groups A and C *Ca*. Hepatoplasma might play roles in the adaptation of offshore dynamics and oligotrophic environments. Group B comprises four ASVs nested among the uncultured bacterium (Bauermeister et al., 2012; Hewson et al., 2018).

## Discussion

### The Gut Microbiota Composition of Ophiuroids

This study characterized the gut microbiota of the Yellow Sea ophiuroids using 16S rRNA sequencing. The taxonomic analysis indicated that the predominant gut microbial phyla were Proteobacteria, Tenericutes, Firmicutes, and Bacteroidetes among the four ophiuroid species. These bacterial phyla have been found in a variety of Echinodermata guts, such as sea cucumbers (Plotieau et al., 2013) and starfish (*Certonardoa semiregularis*) (Lee et al., 2018), commonly constituting over 93.7% of 16S rRNA reads. In addition, the further classification of the largest phylum of Proteobacteria to the class of Gammaproteobacteria showed that this lineage comprised an average of 43.8% (arranging from 26.6 to 60.1%) of ophiuroid gut communities. This ecologically important class has been reported in both the foregut and hindgut of the sea cucumber *Apostichopus japonicus* (Gao et al., 2014), with functions of digesting and degrading debris organisms (Kang et al., 2017).

Previous studies showed the multiple associations of gut microflora with the surrounding environments (Fietz et al., 2018) and hosts (Huang et al., 2020), especially for herbivore feeders. The study of the Pacific white shrimps indicated that the bacterial compositions are almost the same between intestine and sediment with different relative abundance (Fan et al., 2019). In ophiuroids, although gut and sediment microbiota shared the same dominant phylum of Proteobacteria, the gut microbiota were relatively independent reflected by the genera-level of PCA and SIMPER analysis (Figure 5 and Supplementary Appendix A3). The species that mainly contributed for diffidence were of genera *Candidatus* Hepatoplasma, *Colwellia*, *Aliivibrio*, *Poseidonibacter*, and *Woeseia* in Proteobacteria. The difference of gut and ambient sediment microbial communities was also illustrated in the sea cucumber using the diversity and PCA (Gao et al., 2014). Since ophiuroids are the keystone species in the pelagic–sediment coupling layer, further investigations of gut microbiota relationships among the particle organic matter in benthic column layer and surface sediment are needed for ecological roles and correlation in brittle stars and echinoderms.

Despite that our samples were collected from different seasons and sites in the Yellow Sea, the possible variation caused by sampling methods might have limited influence on the composition of gut microbiome. A previous microbial study of sediments in the Northwest Yellow Sea revealed the stable microbial community structure and its subtle seasonal changes (Li, 2009), indicating the minor influence of seasonal factor on the gut microbiota of benthic organisms in the Yellow Sea. In addition, in this study, the microbes of sediment from nearshore and offshore environments were assembled as a whole group by PCA (Figure 5), showing the slight influence of different locations on sediment microbiota. Considering the feeding behavior and living environment of ophiuroids, the stable community structure of the environmental microbiota may provide a relatively steady microenvironment for ophiuroids in this study. However, further annual observation and sequencing studies are still needed for better understanding of the association and interaction between the gut composition of ophiuroids and their environmental factors.

### The Gut Microbiota With the Potential Functions in Ophiuroids

Host phylogeny and dieting habits all contribute to variation in the gut microbiota composition. Studies have shown that the gut microbiota of animals are species-specific and have various biological functions, depending on the feeding types of hosts, including herbivores, carnivores, and omnivores (Gaulke et al., 2018; Huang et al., 2020; Yukgehnaish et al., 2020). Animals with similar feeding habits have a more similar community structure of their intestinal microbiota (Guo et al., 2020), while the composition and functions of gut microbiomes varied among different feeding types (Liu et al., 2016; Wei et al., 2018). The results of various feeding types of fish showed that certain energy metabolism pathways were enriched in herbivore/omnivore and zooplanktivore/zoobenthivore fishes, whereas Lipid metabolism and glycan metabolism were enriched in zoobenthivore/piscivore fishes (Huang et al., 2020).

In this study, the Tax4fun and PICRUSt were used to analyze the functional annotation for different cohorts of ophiuroids, but the consistent results of two methods were obtained only in *S. sladeni* from two sampling sites. The remarkably enriched KEGG pathways belonged to Environmental Information Processing. Given that the higher temperature dynamics in nearshore area and stable bottom water temperature (annual < 10°C annual) of the offshore environment, the overexpression of these environmental pathways in *S. sladeni* might drive their broad-thermal tolerance. The differences of Tax4fun and PICRUSt analysis were commonly reported in microbial studies (Koo et al., 2017; Berlanga et al., 2018), while their variation might be caused by the algorithm for unknown gene prediction (i.e., ancestral state reconstruction algorithm in PICRUSt) and reference database (i.e., Silva-Tax4Fun and Greengenes-PICRUSt; Aßhauer et al., 2015). Further study is essential to integrate the environmental and biological data for refining the microbial functions obtained from analytical methods.

In PICRUSt, server energy metabolism pathways were primarily in abundance in the three scavenger feeders, while the Lipid metabolism and glycan metabolism pathways were enriched in suspension feeder. The KEGG results suggested that the key metabolism pathways associated with growth and development showed high expression in gut microbiota of ophiuroids (Supplementary Appendix A5, A6). The comparison of two nearshore species *O. mirabilis* and *S. sladeni*, belonging to different feeding types, showed significant variations in some nutrient metabolism pathways (Figure 8). The suspension feeder *O. mirabilis* displayed higher-level expression of Carbohydrate metabolism than that in the scavenger feeder *S. sladeni* (Adarsha et al., 2018). The functional difference was likely caused by the genus *Lentibacter* and class Bacteroidia (Figure 4). The genus *Lentibacter* is an aerobic Gram-negative bacterium with association with algae bloom, and their function in hydrocarbon degradation is facilitating the digestion and uptake of organic matter (Angelova, 2017). The class of Bacteroidia, the predominant microbes in *O. mirabilis*, is a member of polysaccharide-degrading consortia (Flint and Duncan, 2014).

As both *S. sladeni* and *O. sarsii vadicola* share close food diets and feeding type as scavenger (Adarsha et al., 2018), the PICRUSt predicted similar expression profiles of metabolism pathways in their gut microbiota. Variation analysis mainly found the significant variations in the environmental adaptation pathways, i.e., Cellular Processes, Genetic Information Processing, and Organismal Systems in two species (Figure 9; Supplementary Appendix A6).

### Candidatus Hepatoplasma: Potential Symbiotic Bacteria and Pathogen of Ophiuroids

Symbiotic bacteria widely identified in the gut microflora from varied marine invertebrates (Carrier and Reitzel, 2020) *Ca.* Hepatoplasma, as a symbiotic bacterium, are comparatively stable intestinal members, which are reported in a variety of the isopod species (Sebastian and Zimmer, 2008; Cheng et al., 2019), sea urchin (Li et al., 2018), and shrimps (Dong et al., 2019). In our study, *Ca*. Hepatoplasma was abundant in the gut of *S. sladeni* from nearshore and offshore environments and existed in *O. kinbergi* and *O. sarsii vadicola*. The BLAST search revealed the low similarity (<50%) of *Ca*. Hepatoplasma between ophiuroids and identified sequences in NCBI. The phylogenetic analysis further revealed the sister relationship between *Ca.* Hepatoplasma in ophiuroids and gut symbionts, such as the genus in amphipods (Figure 10). In amphipods, the genus *Ca*. Hepatoplasma plays an important role in improving growth rate and survival of host under low nutrient conditions (Sebastian and Zimmer, 2008; Horváthová et al., 2015). Due to the high abundance of *Ca.* Hepatoplasma in ophiuroid guts, the association of symbiotic bacteria may improve the nutrition metabolism and survival of brittle stars. Further studies are needed to characterize these symbiotic bacteria and their potential roles of the metabolism and environmental adaptation in ophiuroids.

## Conclusion

Here, we report the gut microbiota of four dominant ophiuroids in the Yellow Sea, and we enhance our understanding of diversity and association of host–microbiomes in the environmental adaptation of echinoderms. Despite the limitations (different times and different locations) of sampling efforts, some interesting phenomena were found in the gut microbiota of ophiuroids between two different feeding types, such as scavenger and suspension feeders. The composition of the gut microbiota exhibited divergent bacterial profiles at higher taxonomic levels in the four ophiuroid species. Functional analysis revealed the significant difference in some metabolism-related pathways, such as Amino acid metabolism and Carbohydrate metabolism, in the herbivores *O. mirabilis* and carnivores *S. sladeni*. Moreover, the putative symbiotic bacteria *Ca*. Hepatoplasma was found in ophiuroid guts, which may facilitate the host’ nutritional metabolism and life-history adaptation. Our work contributed a comprehensive set of data for further understanding the diversity and function of gut microbiota. Further analysis by metagenome and other relative technologies of ophiuroids and their symbiotic bacteria should be conducted to elucidate the host-microbiome interaction and their implication in benthic–pelagic coupling.

## Data Availability Statement

The datasets presented in this study can be found in online repositories. The names of the repository/repositories and accession number(s) can be found below: https://www.ncbi.nlm.nih.gov/, SUB8304440.

## Author Contributions

QX, ZW, and XZ contributed to the conception and design of the study. SF and ZZ collected the samples. YD extracted the DNA. YD and YL performed the statistical analysis. YD, YL, PH, and QX drafted the manuscript. All authors contributed to the manuscript revision and approval of the submitted version.

## Conflict of Interest

The authors declare that the research was conducted in the absence of any commercial or financial relationships that could be construed as a potential conflict of interest.

## Footnotes

1 http://huttenhower.sph.harvard.edu/galaxy
