# Supplementary material for: Gut Microbial Composition and Diversity in Four Ophiuroid Species: Divergence Between Suspension Feeder and Scavenger and Their Symbiotic Microbes
Source: Front Microbiol. 2021 Mar 19;12:645070. doi: 10.3389/fmicb.2021.645070 (PMC8017295; doi:10.3389/fmicb.2021.645070)
Supplement: Supplementary file 2 [file Table_1.DOC]

**Appendix A1**. The number of raw reads, clean reads and amplicon sequence variants (ASVs) in ophiuroids.

| Ophiuroids | | Raw reads | Clean reads | ASVs |
| --- | --- | --- | --- | --- |
| Nearshore | *O. kinbergi* | 19034 | 9101 | 277 |
|  |  | 42098 | 24904 | 496 |
|  |  | 35017 | 20799 | 298 |
|  |  | 41461 | 23955 | 699 |
|  |  | 44453 | 27323 | 576 |
|  | *O. mirabilis* | 47187 | 24955 | 842 |
|  |  | 40786 | 23231 | 764 |
|  |  | 32944 | 17514 | 244 |
|  |  | 46877 | 26468 | 880 |
|  |  | 25289 | 11032 | 365 |
|  | *S. sladeni* | 39033 | 27573 | 118 |
|  |  | 37345 | 24236 | 440 |
|  |  | 27352 | 17341 | 143 |
|  |  | 49633 | 29369 | 444 |
| Offshore | *S. sladeni* | 45761 | 31352 | 181 |
|  |  | 45933 | 28743 | 242 |
|  |  | 33968 | 22515 | 190 |
|  |  | 51521 | 32345 | 267 |
|  |  | 53025 | 34529 | 243 |
|  | *O. sarsii vadicola* | 66339 | 36825 | 314 |
|  |  | 42601 | 26782 | 223 |
|  |  | 54979 | 32252 | 287 |
|  |  | 57227 | 33110 | 346 |

**Appendix A2.** Analysis of similarities of gut microbiota communities from ophiuroids and sediments.

| Sites | Ophiuroid & Sediment | ANOSIM | | PERMANOVA | |
| --- | --- | --- | --- | --- | --- |
|  |  | R | *p* | R | *p* |
| Nearshore | *S. sladeni &* Sediment | 0.278 | 0.086 | 1.7003 | 0.0854 |
|  | *O. mirabilis &* Sediment | 0.231 | 0.125 | 1.6287 | 0.1112 |
|  | *O. kinbergi &* Sediment | 0.251 | 0.125 | 2.4198 | 0.0737 |
| Offshore | *S. sladeni &* Sediment | 0.940 | 0.008 | 18.128 | 0.0078 |
|  | *O. sarsii vadicola &* Sediment | 1.000 | 0.008 | 21.977 | 0.0072 |

***Appendix A3.*** *Similarity percentages analysis of gut microbiota communities of* *S. sladeni,* *O. mirabilis and O. kinbergi.*

| Group | Av.Abund | | Av.Diss% | Contrib% | Cum.% |
| --- | --- | --- | --- | --- | --- |
|  | *S. sladeni* | *O. mirabilis* |  |  |  |
| NA | 0.63 | 0.47 | 15.99 | 29.49 | 29.49 |
| *Ca.* Hepatoplasma | 0.18 | 0.00 | 9.04 | 16.68 | 46.17 |
| *Lentibacter* | 0.00 | 0.08 | 4.05 | 7.47 | 53.64 |
| *Anoxybacillus* | 0.02 | 0.01 | 1.23 | 2.26 | 55.90 |
| *Salinispira* | 0.02 | 0.00 | 1.18 | 2.17 | 58.06 |
| *Woeseia* | 0.00 | 0.02 | 1.05 | 1.93 | 59.99 |
| *Anaerobacillus* | 0.02 | 0.03 | 0.88 | 1.63 | 61.62 |
| *Subgroup 10* | 0.00 | 0.02 | 0.85 | 1.57 | 63.19 |
| *Dubosiella* | 0.00 | 0.02 | 0.75 | 1.39 | 64.58 |
| *Lachnospiraceae NK4A136 group* | 0.00 | 0.01 | 0.65 | 1.21 | 65.78 |
| *Akkermansia* | 0.00 | 0.01 | 0.64 | 1.18 | 66.96 |
| *Bacillus* | 0.01 | 0.02 | 0.58 | 1.06 | 68.03 |
| *Methyloversatilis* | 0.00 | 0.01 | 0.55 | 1.02 | 69.04 |
|  | *S. sladeni* | *O. kinbergi* |  |  |  |
| NA | 0.63 | 0.41 | 17.47 | 30.71 | 30.71 |
| *Ca.* Hepatoplasma | 0.18 | 0.04 | 9.18 | 16.13 | 46.84 |
| *Photobacterium* | 0.00 | 0.09 | 4.31 | 7.57 | 54.41 |
| *Vibrio* | 0.01 | 0.07 | 3.26 | 5.73 | 60.14 |
| *Ca.* Thiophysa | 0.00 | 0.04 | 2.13 | 3.74 | 63.88 |
| *Anoxybacillus* | 0.02 | 0.04 | 2.09 | 3.67 | 67.55 |
| *Salinispira* | 0.02 | 0.00 | 1.18 | 2.07 | 69.62 |
| *Pseudoalteromonas* | 0.00 | 0.02 | 0.67 | 1.17 | 70.79 |
|  | *O. mirabilis* | *O. kinbergi* |  |  |  |
| NA | 0.47 | 0.41 | 8.97 | 17.27 | 17.27 |
| *Photobacterium* | 0.01 | 0.09 | 4.25 | 8.19 | 25.45 |
| *Lentibacter* | 0.08 | 0.00 | 4.04 | 7.77 | 33.22 |
| *Vibrio* | 0.01 | 0.07 | 3.16 | 6.07 | 39.29 |
| *Ca.* Thiophysa | 0.00 | 0.04 | 2.13 | 4.09 | 43.39 |
| *Anoxybacillus* | 0.01 | 0.04 | 1.82 | 3.51 | 46.90 |
| *Ca.* Hepatoplasma | 0.00 | 0.04 | 1.72 | 3.30 | 50.20 |
| *Anaerobacillus* | 0.03 | 0.02 | 0.97 | 1.87 | 52.07 |
| *Dubosiella* | 0.02 | 0.01 | 0.89 | 1.72 | 53.79 |
| *Lachnospiraceae NK4A136 group* | 0.01 | 0.01 | 0.79 | 1.53 | 55.32 |
| *Subgroup 10* | 0.02 | 0.01 | 0.72 | 1.39 | 56.71 |
| *Woeseia* | 0.02 | 0.02 | 0.69 | 1.33 | 58.04 |
| *Akkermansia* | 0.01 | 0.00 | 0.68 | 1.31 | 59.35 |
| *Pseudoalteromonas* | 0.00 | 0.02 | 0.65 | 1.25 | 60.60 |
| *Aliivibrio* | 0.01 | 0.01 | 0.65 | 1.25 | 61.85 |
| *Bacillus* | 0.02 | 0.01 | 0.60 | 1.16 | 63.01 |
| *Methyloversatilis* | 0.01 | 0.00 | 0.54 | 1.04 | 64.05 |
| *BD1-7 clade* | 0.00 | 0.01 | 0.53 | 1.02 | 65.07 |

**Appendix A4.** Cladogram plot by LDA Effect Size (LEfSe) analysis of gut microbiota in ophiuroids. Y, the nearshore environment; H, the offshore environment; f, the family; o, the order; c, the class; p, the phylum.


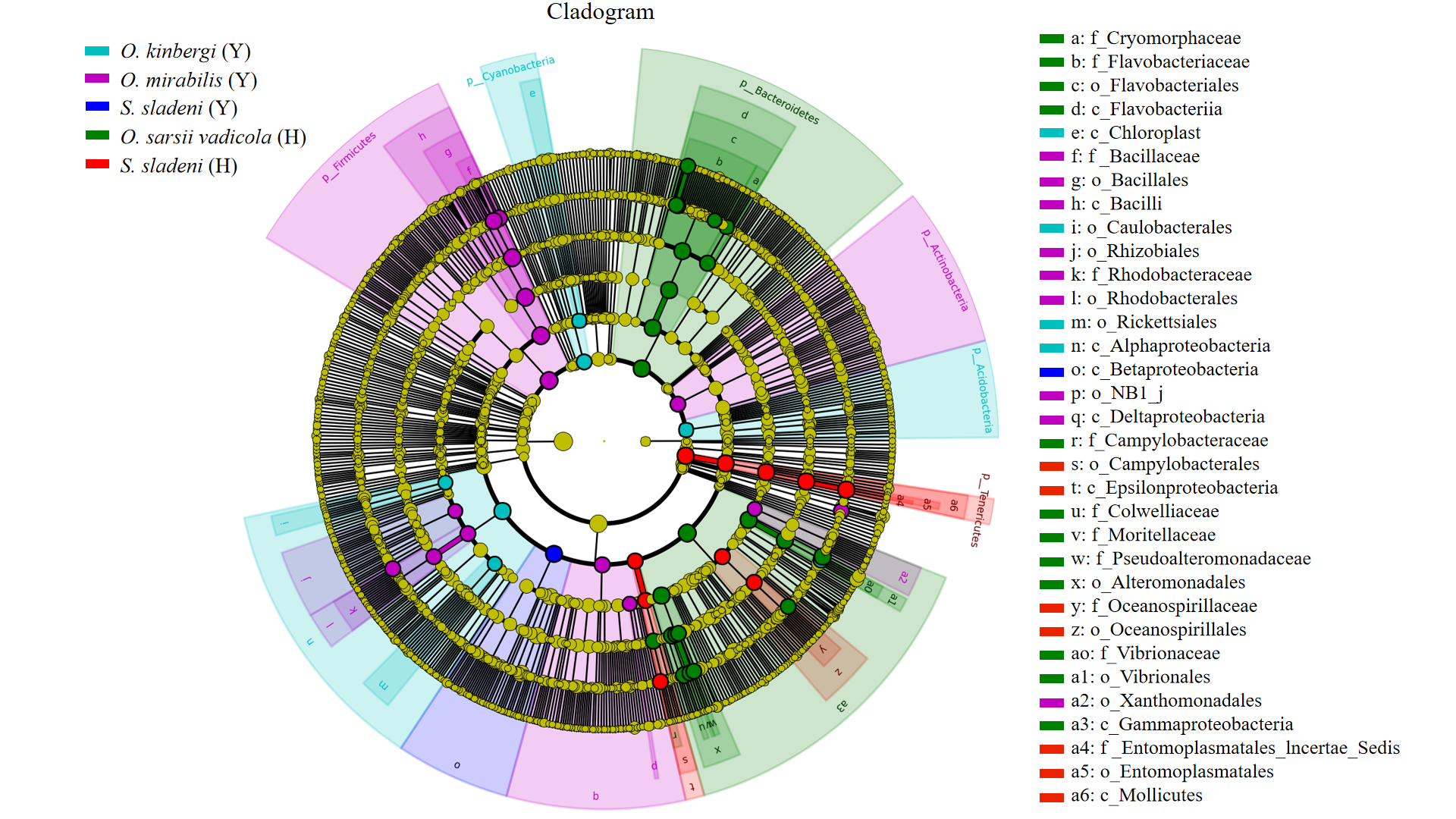


**Appendix A5.** The heatmap of Kyoto encyclopedia of genes and genomes (KEGG) ortholog (KOs) groups predicted by Tax4Fun analysis in ophiuroids at KEGG level 2. Y, the nearshore environment; H, the offshore environment.





**Appendix A6.** The heatmap of Kyoto encyclopedia of genes and genomes (KEGG) ortholog (KOs) groups predicted by PICRUSt analysis in ophiuroids at KEGG level 2. Y, the nearshore environment; H, the offshore environment.

**Appendix A7.** Variation analysis of gut microbial community between *S. sladeni* and *O. mirabilis* from nearshore environment at Kyoto Encyclopedia of Genes and Genomes level 2 by Tax4Fun.


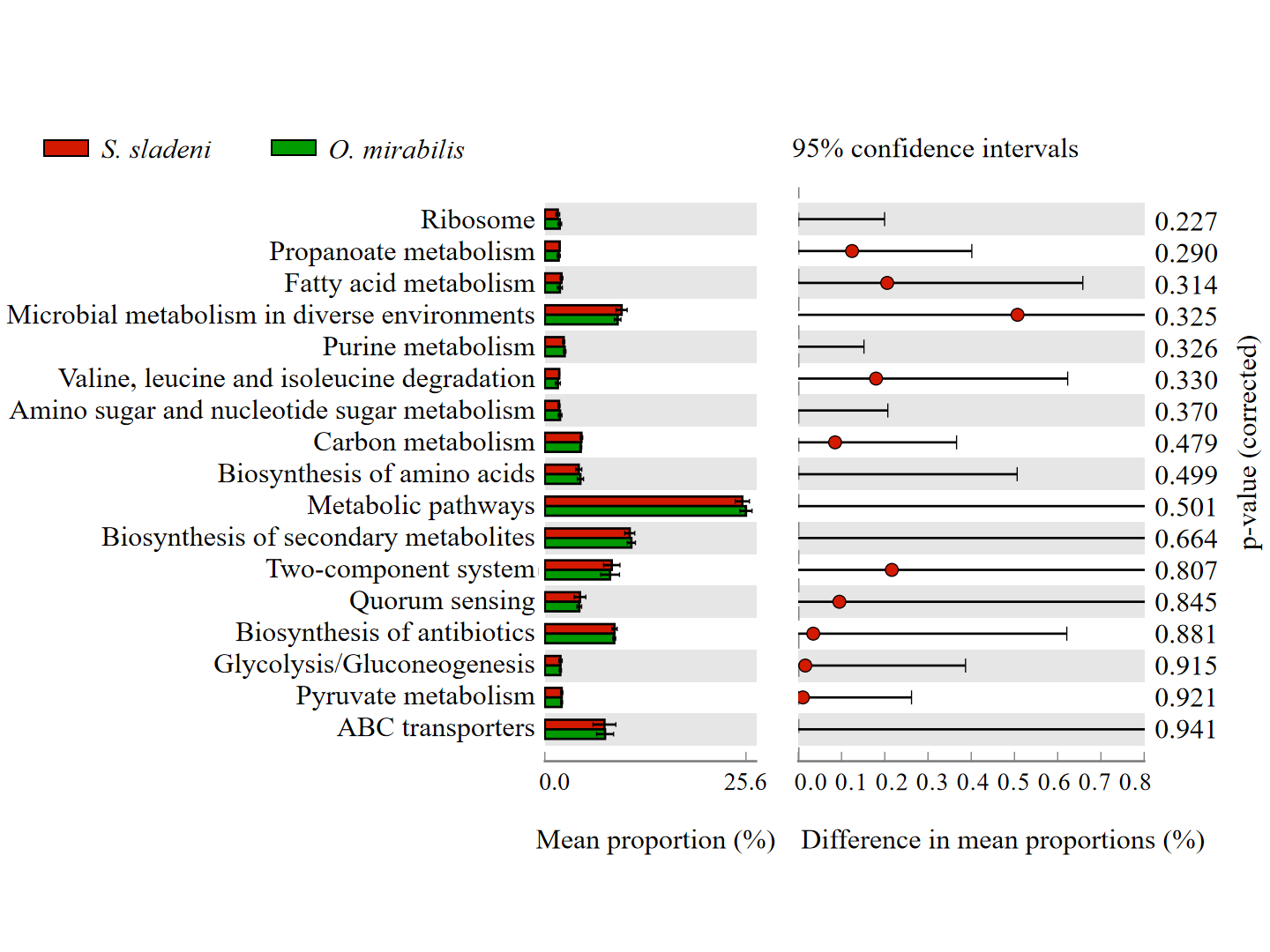


**Appendix A8.** Variation analysis of gut microbial community between *S. sladeni* and *O. sarsii vadicola* from offshore environment at Kyoto Encyclopedia of Genes and Genomes level 2 by Tax4Fun.


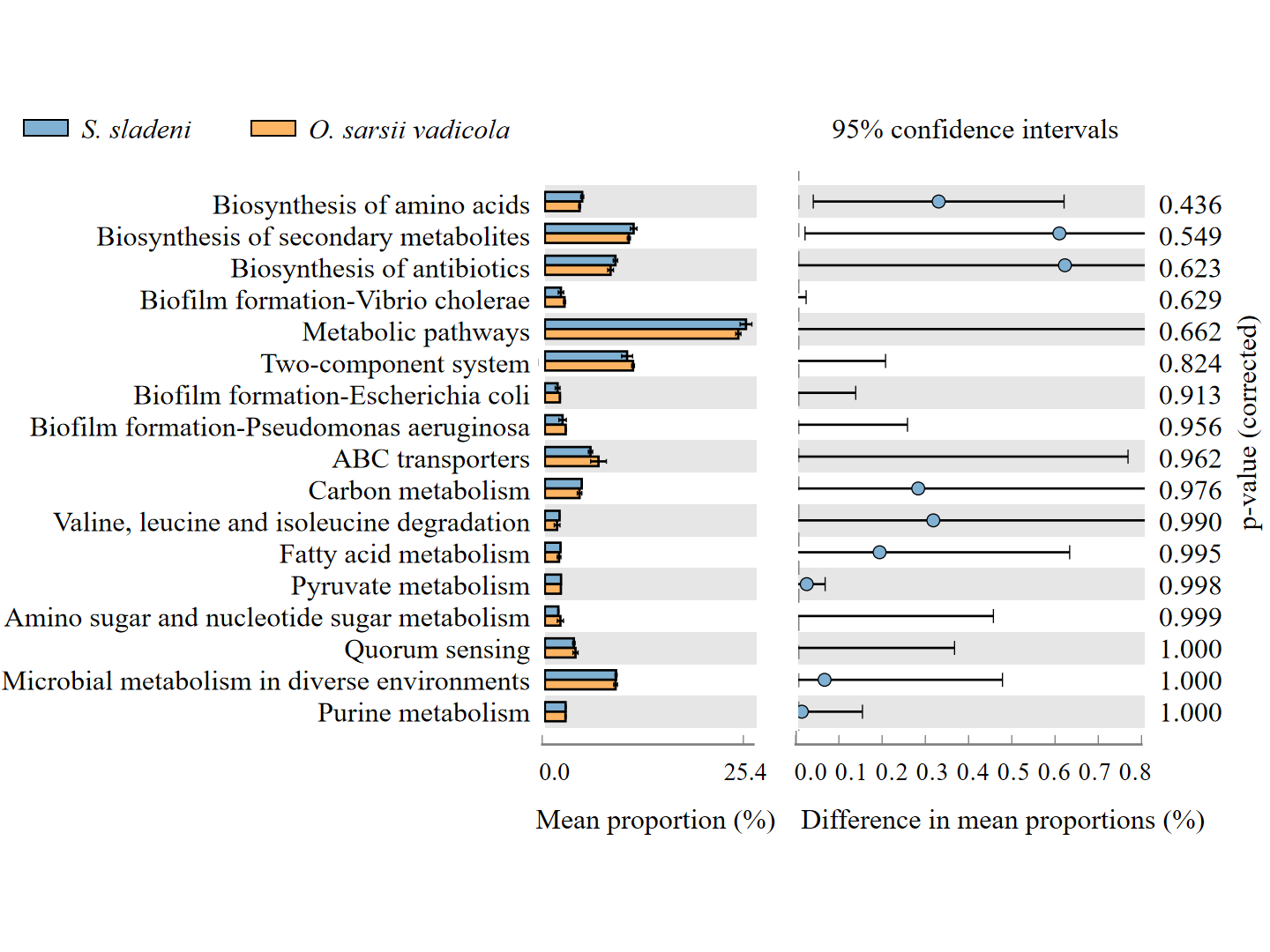


**Appendix A9.** ASV number, top hit, Identity, Class classification, GenBank number and references of homologous sequences with BLASTn identity > 80% and E-value of 1e-20 in Figure 10.

| ASV | Top hit | Identity | Class | GenBank | Reference |
| --- | --- | --- | --- | --- | --- |
| ASV1 | Uncultured microorganism clone RS 9-48 16S ribosomal RNA gene | 89.93% | unclassified sequences | MG518611 | Hewson et al., (2018) |
| ASV8 | Uncultured microorganism clone RS 9-48 16S ribosomal RNA gene | 89.71% | unclassified sequences | MG518611 | Hewson et al., (2018) |
| ASV20 | Uncultured bacterium clone BUG084 28 16S ribosomal RNA gene | 90.16% | - | JN983627 | Bauermeister et al., (2012) |
| ASV25 | Uncultured Mollicutes bacterium clone Cobs2TisF5 16S ribosomal RNA gene | 92.19% | Mollicutes | EU246820 | Speck M D et al., (2007), unpublished |
| ASV46 | Uncultured bacterium clone BUG084 28 16S ribosomal RNA gene | 89.93% | - | JN983627 | Bauermeister et al., (2012) |
| ASV112 | Uncultured Mollicutes bacterium gene for 16S ribosomal RNA | 91.48% | Mollicutes | AB980074 | Watsuji et al., (2015) |
| ASV161 | Uncultured bacterium partial 16S rRNA gene, clone LBDT3651-B | 99.10% | - | HE663395 | Duperron et al., (2013) |

**Appendix A10.** Class classification, source, GenBank number and references of sequence from different host.

| Class | Source | GenBank | Reference |
| --- | --- | --- | --- |
| Mollicutes | uncultured Mollicutes bacterium | FM863735 | Durand et al., (2009) |
| Mollicutes | Mycoplasma microti | U22415 | Dillehay et al., (1995) |
| Mollicutes | uncultured Mycoplasma sp. | KC169759 | Green et al., (2013) |
| Mollicutes | uncultured Mycoplasmataceae bacterium | AY539724 | Wang et al., (2007) |
| Mollicutes | *Ca*. Hepatoplasma crinochetorum | AY500250 | Wang et al., (2004) |
| Mollicutes | uncultured Mycoplasmataceae bacterium | EU646191 | Sebastian, Zimmer (2008) |
| Mollicutes | uncultured Mycoplasmataceae bacterium | KC918290 | Givens et al., (2013) |
| Mollicutes | uncultured Mollicutes bacterium | FR839078 | Durand et al., (2015) |
| Mollicutes | *Ca*. Hepatoplasma | SRR8517657 | Cheng et al., (2019) |

**REFERENCE**

Bauermeister J, Ramette A, Dattagupta S. 2012. Repeatedly evolved host-specific ectosymbioses between sulfur-oxidizing bacteria and amphipods living in a cave ecosystem. *Plos One*, **7** (11): e50254.

Cheng X, Wang Y, Li J, Yan G, He L. 2019. Comparative analysis of the gut microbial communities between two dominant amphipods from the Challenger Deep, Mariana Trench. *Deep Sea Research Part I: Oceanographic Research Papers*, **151** 103081.

Dillehay D L, Sander M, Talkington D F, Thacker W, Brown D R. 1995. Isolation of mycoplasmas from prairie voles (*Microtus ochrogaster*). *Laboratory Animal Science*, **45** (6): 631-634.

Duperron S, Pottier M-A, Leger N, Gaudron S M, Puillandre N, Prieur S L, Sigwart J D, Ravaux J, Zbinden M. 2013. A tale of two chitons: is habitat specialisation linked to distinct associated bacterial communities? *FEMS Microbiology Ecology*, **83** (3): 552-567.

Durand L, Roumagnac M, Cueff-Gauchard V, Jan C, Guri M. 2015. Biogeographical distribution of *Rimicaris exoculata* resident gut epibiont communities along the Mid-Atlantic Ridge hydrothermal vent sites. *FEMS Microbiology Ecology*, **91** (10):

Durand L, Zbinden M, Cueff-Gauchard V, Duperron S, Roussel E G, Shillito B, Cambon-Bonavita M-A. 2009. Microbial diversity associated with the hydrothermal shrimp *Rimicaris exoculata* gut and occurrence of a resident microbial community. *FEMS Microbiology Ecology*, **71** (2): 291-303.

Givens C E, Burnett K G, Burnett L E, Hollibaugh J T. 2013. Microbial communities of the carapace, gut, and hemolymph of the Atlantic blue crab, *Callinectes sapidus*. *Marine Biology*, **160** (11): 2841-2851.

Green T J, Smullen R, Barnes A C. 2013. Dietary soybean protein concentrate-induced intestinal disorder in marine farmed Atlantic salmon, *Salmo salar* is associated with alterations in gut microbiota. *Veterinary Microbiology*, **166** (1-2): 286-292.

Hewson I, Bistolas K S, Cardé E M Q, Button J B, Foster P J, Flanzenbaum J M, Kocian J, Lewis C K. 2018. Investigating the complex association between viral ecology, environment, and northeast Pacific sea star wasting. *Frontiers in Marine Science*, **5** 77.

Sebastian F, Zimmer M. 2008. Host-specificity of environmentally transmitted Mycoplasma-like isopod symbionts. *Environmental Microbiology*, **10** (10): 2497-2504.

Wang Y, Brune A, Zimmer M. 2007. Bacterial symbionts in the hepatopancreas of isopods: diversity and environmental transmission. *FEMS Microbiology Ecology*, **61** (1): 141-152.

Wang Y, Stingl U, Anton-Erxleben F, Geisler S, Brune A, Zimmer M. 2004. “*Candidatus* Hepatoplasma crinochetorum,” a new, stalk-forming lineage of *Mollicutes* colonizing the midgut glands of a terrestrial isopod. *Applied and Environmental Microbiology*, **70** (10): 6166-6172.

Watsuji T-o, Yamamoto A, Motoki K, Ueda K, Hada E, Takaki Y, Kawagucci S, Takai K. 2015. Molecular evidence of digestion and absorption of epibiotic bacterial community by deep-sea crab Shinkaia crosnieri. *The ISME Journal*, **9** (4): 821-831.
